# Supplementary material for: Human monoclonal antibodies to HPV16 show evidence for common developmental pathways and public epitopes
Source: PLoS Pathog. 2025 Oct 21;21(10):e1013086. doi: 10.1371/journal.ppat.1013086 (PMC12551957; doi:10.1371/journal.ppat.1013086)
Supplement: S1 Table — Antibodies in this manuscript (column 1) are cross referenced to identifiers used in Scherer et. al. 2018 [22] (column 2). The first letter in the current ID represents the study subject, the following number is the month at which the sample was collected. If the number is a decimal it was collected one week after the vaccine dose. The letter M or P indicates the source was a memory B cells or plasmablast and last two digits were added to create unique identifier. The neutralization titer (IC50) units are picomolar (pM). (PDF) [file ppat.1013086.s001.pdf]

S1 Table Antibody cross-reference.

Current ID	Previous ID	Accession HC	Accession LC	IC50 (pM)
A7M01	HPV16.55	PV796198	PV796199	1.60
A7M05	HPV16.57	PV796200	PV796201	0.20
A7M08	HPV16.58	PV796202	PV796203	3.30
A7M11	HPV16.61	PV796204	PV796205	0.20
A7M13	HPV16.63	PV796206	PV796207	3.40
A7M15	HPV16.64	PV796208	PV796209	5.10
A7M18	HPV16.66	PV796210	PV796211	38.10
A24M02	HPV16.96	PV796186	PV796187	5.34
A24M03	HPV16.97	PV796188	PV796189	0.96
A24M04	HPV16.99	PV796190	PV796191	8.50
A24M06	HPV16.166	PV796192	PV796193	30.70
A24.1M02	HPV16.104	PV796184	PV796185	9.54
A25M02	HPV16.129	PV796194	PV796195	30.25
A25M03	HPV16.130	PV796196	PV796197	6.80
B6.1P01	HPV16.91	PV796182	PV796183	12.45
B7M06	HPV16.69	PV796226	PV796227	1.70
B7M07	HPV16.70	PV796228	PV796229	14.70
B7M14	HPV16.71	PV796230	PV796231	6.70
B24M01	HPV16.100	PV796216	PV796217	36.00
B24.1P01	HPV16.108	PV796180	PV796181	11.24
B24.1M01	HPV16.105	PV796212	PV796213	29.99
B24.1M03	HPV16.107	PV796214	PV796215	5.50
B25M01	HPV16.131	PV796218	PV796219	15.90
B25M02	HPV16.132	PV796220	PV796221	21.20
B25M05	HPV16.135	PV796222	PV796223	3.04
B25M06	HPV16.136	PV796224	PV796225	2.20
D6.1M01	HPV16.94	PV796274	PV796275	1.01
D6.1M02	HPV16.167	PV796276	PV796277	15.10
D6M02	HPV16.84	PV796278	PV796279	6.17
D7M01	HPV16.90	PV796280	PV796281	1.60
D24.1M01	HPV16.111	PV796232	PV796233	14.21
D24.1M02	HPV16.112	PV796234	PV796235	19.63
D24.1M06	HPV16.116	PV796236	PV796237	0.55
D24.1M07	HPV16.117	PV796238	PV796239	1.74
D24.1M08	HPV16.118	PV796240	PV796241	10.86
D24.1M09	HPV16.119	PV796242	PV796243	3.66
D24.1M10	HPV16.120	PV796244	PV796245	1.68
D24.1M11	HPV16.121	PV796246	PV796247	3.79
D24.1M12	HPV16.168	PV796248	PV796249	8.10
D25M01	HPV16.137	PV796250	PV796251	7.02
D25M02	HPV16.138	PV796252	PV796253	23.40
D25M03	HPV16.139	PV796254	PV796255	75.60
D25M05	HPV16.141	PV796256	PV796257	1.14
D25M07	HPV16.143	PV796258	PV796259	0.69
D25M08	HPV16.144	PV796260	PV796261	0.88
D25M10	HPV16.145	PV796262	PV796263	6.83
D25M11	HPV16.147	PV796264	PV796265	0.89
D25M12	HPV16.149	PV796266	PV796267	117.20
D25M13	HPV16.148	PV796268	PV796269	16.88
D25M15	HPV16.151	PV796270	PV796271	6.00
D25M16	HPV16.150	PV796272	PV796273	5.00
E7M03	HPV16.73	PV796314	PV796315	1.80
E24.1M01	HPV16.122	PV796282	PV796283	14.60
E24.1M02	HPV16.123	PV796284	PV796285	34.80
E24.1M03	HPV16.124	PV796286	PV796287	10.30
E24.1M05	HPV16.126	PV796288	PV796289	2.67
E24.1M06	HPV16.127	PV796290	PV796291	4.04
E25M02	HPV16.154	PV796292	PV796293	0.78
E25M03	HPV16.155	PV796294	PV796295	11.55
E25M04	HPV16.156	PV796296	PV796297	6676.00
E25M05	HPV16.157	PV796298	PV796299	24.30
E25M06	HPV16.158	PV796300	PV796301	1.92
E25M07	HPV16.159	PV796302	PV796303	0.96
E25M08	HPV16.160	PV796304	PV796305	66.97
E25M09	HPV16.161	PV796306	PV796307	8.09
E25M10	HPV16.162	PV796308	PV796309	2.80
E25M11	HPV16.163	PV796310	PV796311	2.88
E25M12	HPV16.164	PV796312	PV796313	0.44
