# Supplementary material for: Human monoclonal antibodies to HPV16 show evidence for common developmental pathways and public epitopes
Source: PLoS Pathog. 2025 Oct 21;21(10):e1013086. doi: 10.1371/journal.ppat.1013086 (PMC12551957; doi:10.1371/journal.ppat.1013086)
Supplement: S2 Table — These are the same data shown in Fig 1B however in this figure the percent neutralization is shown. Antibodies were tested in triplicate in neutralization assays against six cpsV, psV16 and psv31 using a concentration of antibody of 100-fold higher than their IC50 versus psV16. Each cpsV had amino acid substitutions on one of the five surface loops of HPV16 as indicated in Fig 1A. The value is percent neutralization: setting psV with no antibody equal to zero and no psV equal to 100%. Clustering was performed manually keep similar neutralization profiles and clonotypes together. Clonotypes are indicated by shading in column 2 and 3. (PDF) [file ppat.1013086.s002.pdf]

S2 Table Determining which loops were required for neutalizaiton.

	IGHV	IGLV	BC	DE	EF	FGa	FGb	HI	16wt	31wt
D24.1M02	2-70	1-40	98.85	102.5	98.78	101.54	100.15	100.61	100.88	-25.71
D25M02	2-70	1-40	98.28	100.63	100.82	102.97	100.19	101.48	100.53	15.43
D24.1M12	2-70	1-40	99.16	100.29	100.29	100.2	100.03	100.37	100.19	9.07
D25M03	2-70	1-40	99.01	101.58	100.67	100.24	100.37	99.97	100.38	28.11
D24.1M01	2-70	1-40	97.42	104.38	103.67	103.2	100.33	100.09	102.12	-1.14
D25M15	2-70	1-40	98.9	99.93	99.35	99.76	99.71	100.54	100.1	9.61
E25M05	1-18	1-39	98.95	100.04	99.94	99.67	99.92	99.88	99.77	-9.47
E25M09	1-18	1-39	98.63	99.96	100.51	99.64	100.1	99.64	100	5.98
E25M04	**2-70**	**1-40**	98.33	100.69	103.22	104.4	100.94	101.2	101.2	10.76
B25M02	**2-70**	**1-40**	99.82	105.27	105.12	102.21	100.44	99.57	105.8	-63.64
A7M15	**2-70**	**1-40**	97.11	101.29	99.93	101.9	102.39	100.72	99.66	-3.9
E25M08	**3-21**	**1-5**	98.82	100.07	100.24	99.06	100.04	100.02	99.95	5.35
E25M03	**4-4**	**1-39**	98.62	99.86	99.41	99.99	96.93	99.91	99.73	-4.32
D24.1M10	**4-39**	**1-39**	98.53	99.4	99.81	99.8	100.12	100.05	100.36	-9.24
E25M12	**4-39**	**1-39**	99.08	88.97	100.13	100.06	96.01	100.07	99.93	1.6
A24M02	**3-49**	**3-21**	96.92	101.18	102.44	90.95	100.31	80.67	102.08	5.16
D24.1M08	4-31	1-39	98.22	99.86	99.8	100.04	100.3	100.58	100.28	-0.4
D25M16	4-31	1-39	98.87	96.84	99.42	98.41	77.27	100.3	99.96	2.62
E25M11	**1-69-2**	**1-6**	99.23	98.23	78.77	100.27	100.56	100.23	100.1	90.54
E24.1M03	**3-30**	**1-44**	96	101.04	102.85	98.22	100.06	75.16	98.76	0
A7M08	**2-70**	**1-40**	21.83	99.28	99.56	100.2	98.14	99.16	100.23	0.96
D7M01	4-34	1-47	85.35	86.96	85.85	85.56	-22.72	73.21	98.71	-1.66
D6M02	4-34	1-47	80.89	79.79	80.85	76.99	-26.49	60.02	98	16.34
D25M08	4-34	1-47	92.52	95.52	92.15	96.54	32.65	90.94	99.26	-4.04
D24.1M06	1-46	3-21	90.29	94.5	98.34	99.25	13.31	98.89	99.9	58.95
D25M07	1-46	3-21	90.92	94.76	95.6	99.1	-0.77	97.18	99.96	41.22
D6.1M01	1-46	1-17	98.38	100.33	102.56	103.81	3.6	99.95	99.96	2.25
D24.1M07	1-46	1-17	98.33	100.24	100.51	100.93	-33.98	100.72	100.42	94.72
D24.1M09	1-46	1-17	96.9	99.98	100.11	100.46	-134.67	99.99	99.99	29.26
D25M11	1-46	1-17	97.76	100.17	102.19	103.71	-25.73	99.81	99.86	-2.32
A24.1M02	**3-23**	**1-39**	98.34	101.68	101.5	102.24	29.3	103.49	100.73	-52.58
B25M06	**3-48**	**1-17**	98.53	99.87	100	99.94	12	100.33	100.41	90.03
B25M05	**4-34**	**2-14**	97.21	99.21	97.36	96.32	30.29	71.58	93	-19.19
D25M10	**4-34**	**1-6**	98.5	99.11	98.25	99.7	0.64	95.57	99.93	89.15
D25M12	**4-34**	**1-17**	98	100.06	102.86	103.85	-5.06	92.02	100.05	9.82
E24.1M01	**5-51**	**4-1**	98.8	99.92	99.62	99.88	2.71	100.28	100.13	4.38
E24.1M02	**3-48**	**3-21**	105.47	101.68	100.66	102.08	-35.67	100.17	99.75	8.72
A7M05	**5-51**	**2-11**	98.08	98.11	99.31	99.62	-181.77	98.6	99.94	32.53
E25M10	**3-21**	**1-6**	98.96	99.72	100.17	99.92	1.02	9.65	100.02	5.47
E25M07	**3-48**	**1-6**	99.02	99.86	99.9	99.86	2.67	29.22	99.82	1.57
E7M03	**4-34**	**3-11**	94.72	-14.33	86.56	93.89	2.28	83.37	90.02	-0.4
D6.1M02	**4-34**	**1-39**	98.68	1.59	99.3	99.59	13.44	98.87	99.27	12.74
A7M01	**3-48**	**3-20**	95.22	-44.45	97.13	96.32	-41.25	93.55	97.35	-31.57
B24.1M01	**4-34**	**2-14**	94.77	-23.06	99.34	86.62	51.86	80.69	95.09	-26.26
B24.1M03	**4-4**	**1-39**	98.22	0.21	99.41	99.21	33.21	100.18	100.28	-0.27
D24.1M11	**4-4**	**1-39**	98.16	-2.01	99.68	99.25	5.65	99.14	99.56	2.95
D25M05	**1-69**	**2-14**	96.24	6.69	102.28	103.39	-18.61	99.1	99.53	-8.44
E25M02	**3-7**	**2-23**	98.48	12.96	102.72	103.92	64.77	100.44	100.12	8.4
A7M11	**4-30-2**	**1-44**	98.03	36.18	100.42	100.38	71.9	99.22	100.27	6.03
A24M03	**3-7**	**1-39**	98.44	-10.75	100.46	100.08	95.29	100.38	99.97	-23.23
E25M06	**1-24**	**1-39**	99.01	-12	99.98	99.96	100.24	99.93	99.87	0.34
E24.1M05	**3-33**	**1-39**	98.68	-1.76	99.83	99.88	100.17	100.07	99.78	4.01
A7M18	1-69	1-40	97.3	99.0	-24.0	96.3	99.0	79.6	93.8	-3.8
A25M02	1-69	1-40	97.1	98.81	-23.6	99.74	99.01	86.99	99.17	11.69
E24.1M06	**3-73**	**1-39**	98.62	99.93	7.75	99.9	100.19	100.12	99.98	-1.1
B6.1P01	3-7	2-14	97.77	99.76	100.78	30.87	98.72	88.49	99.46	7.7
B24M01	3-7	2-14	97.51	99.14	100.35	30.21	98.32	86.43	99.31	5.16
B24.1P01	3-7	2-14	96.76	99.62	100.77	-4.51	98.9	87.65	99.54	-0.76
B7M06	3-9	1-39	93.84	98.22	90.79	76.02	82.77	39.92	101.35	1.03
B7M07	3-9	1-39	96.76	101.68	99.81	98.42	97.9	23.23	103.06	-14.44
B7M14	3-9	1-39	98.82	102.37	105.08	100.41	97.1	11.19	100.36	-28.87
A24M04	**3-9**	**1-39**	98.38	99.96	100.19	100.12	100.18	4.99	100	-2.88
A7M13	**3-23**	**1-9**	99.12	99.93	100.33	99.95	99.96	3.09	99.88	6.16
A24M06	**3-43**	**1-12**	99.13	100.02	99.71	1.95	99.48	11.53	100.31	11.99
A25M03	**3-30**	**1-17**	98.15	99.91	99.94	30.7	100.18	55.59	100.58	1.34
D25M01	**3-23**	**1-27**	96.86	100.08	92.03	7.35	79.3	-1.22	99.35	-8.75
B25M01	**3-30**	**3-11**	100	104.35	-12.7	102.65	99.94	13.02	105.65	-56.57
D25M13	**4-61**	**1-5**	98.5	98.89	0.41	85.35	15.52	-3.75	99.87	-1.46

10	20	30	40	50	60	70	80	90	
