# Supplementary material for: Human monoclonal antibodies to HPV16 show evidence for common developmental pathways and public epitopes
Source: PLoS Pathog. 2025 Oct 21;21(10):e1013086. doi: 10.1371/journal.ppat.1013086 (PMC12551957; doi:10.1371/journal.ppat.1013086)
Supplement: S3 Table — The germline CDRL2 sequence was cloned into the light chains for each mAb listed. The modified light chains were coexpressed with their cognate heavy chain and used in neutralization assays with psV16. The fold differences are compared with results using the mature light chain for each (see sequence Fig 3C). (PDF) [file ppat.1013086.s003.pdf]

S3 Table Measuring the importance of the CDRL2 region for neutralization of psV16.

Antibody	Germline CDRL2*	Mature CDRL2	difference	P value[1]
A7M08	1.12×10^5 +/- 2.45×10^4	12.0 +/- 5.5	9346	**> 0.001**
A7M15	228.7 +/- 17.3	33.0 +/- 1.7	6.9	**> 0.001**
D24.1M01	119.3 +/- 18.0	22.7 +/- 4.5	5.3	**> 0.001**
D24.1M02	451.3 +/- 71.3	20.3 +/- 6.7	22.2	**> 0.001**
E25M04	2.70×10^4 +/- 2.3×10^4	680 +/- 399	39.7	**0.006**
B25M02	21.5 +/- 3.2	18.7 +/- 3.7	1.14	0.39
D25M03	72.0 +/- 5.9	76.7 +/- 7.4	0.94	0.44

* IC_{50} pM +/- SD. [1]Students t-test
