# Supplementary material for: Human monoclonal antibodies to HPV16 show evidence for common developmental pathways and public epitopes
Source: PLoS Pathog. 2025 Oct 21;21(10):e1013086. doi: 10.1371/journal.ppat.1013086 (PMC12551957; doi:10.1371/journal.ppat.1013086)
Supplement: S4 Table — Data collection information for the cryo-EM data and statistics from refinement of the models. MolProbity [71] and EMRinger [72] were used for analysis of the quality of the final model. (PDF) [file ppat.1013086.s004.pdf]

S4 Table Data collection and refinement statistics for cryo-EM structures, related to Fig. 4 and 6

	D24M01 Fab with HPV16 L1	A7M08 Fab with HPV16 L1	B25M05 Fab with HPV16 L1
**Data collection**			
Microscope	Krios	Glacios	Glacios
Voltage (kV)	300	200	200
Electron Dose (e-/Å^2)	50	51	51
Detector	3EC	K3	K3
Pixel Size (Å/px)	1.054	1.122	1.122
Defocus Range (µm)	-0.8 to -2.2	-1.5 to -2.2	-1.8 to -2.2
Collection Tilt (°)	0	0, 30, 45	0
Magnification	96,000x	36,000x	36,000x
**Reconstruction**			
Software	CryoSPARC v3.1	CryoSPARC v4.4	CryoSPARC v4.4
Selected Micrographs	2,332	1,584	753
Selected Particles	188,743	301,713	83,711
Symmetry	C5	C5	C1
Box Size (px)	220	320	320
Resolution (Å) ($FSC_{0.143}$)			
Unmasked	3.3	3.7	3.7
Masked	3.0	2.7	2.9
**Refinement**			
Map B factor (Å^2)	176.5	130.4	128.5
No. atoms			
Protein	50,319	46,480	35,628
Water	0	0	0
Ligand	0	0	0
Mean B-factor (Å)			
Protein	63.00	44.80	99.99
Water	0	0	0
Ligand	0	0	0
RMS bond length (Å)	0.005	0.006	0.004
RMS bond angle (°)	0.699	0.613	0.540
**Validation**			
MolProbity	2.34	1.69	1.18
Clashscore	6.11	3.31	1.99
CaBLAM outliers (%)	2.7	2.86	1.5
EMRinger	2.62	2.83	3.93
Rotamer Outliers (%)	0.75	1.73	0.25
Ramachandran			
Favored (%)	94.49	94.26	96.66
Disallowed (%)	0.15	0.00	0.00
Average Q score	0.41	0.53	0.56
**PDB ID**	**9ML1**	**9ML2**	**9ML3**
**EMDB ID**	**48344**	**48345**	**48346**
