# Supplementary material for: Human monoclonal antibodies to HPV16 show evidence for common developmental pathways and public epitopes
Source: PLoS Pathog. 2025 Oct 21;21(10):e1013086. doi: 10.1371/journal.ppat.1013086 (PMC12551957; doi:10.1371/journal.ppat.1013086)

S1 Fig. Human mAbs potently neutralize HPV16 in vitro.

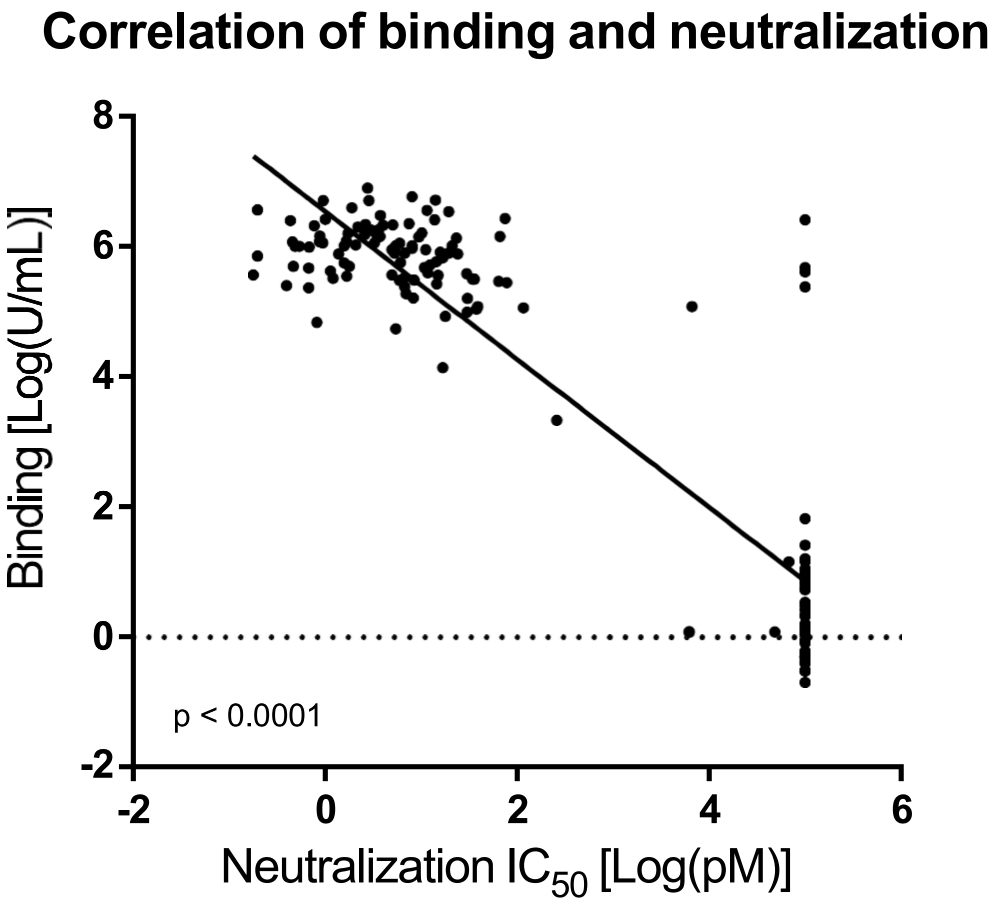

Supplement: S1 Fig — 148 hmAb, including the 68 mAb included in this study were tested for HPV16 neutralization and binding. Antibodies that failed to neutralize were given an arbitrary IC50 of 100 nM. There were four mAb (circled) that had significant binding but failed to neutralize. The p value is from linear regression, indicating that the slope of the line is statistically significantly not equal to zero. (PDF) [file ppat.1013086.s005.pdf]
