# Supplementary figures and images for: Human monoclonal antibodies to HPV16 show evidence for common developmental pathways and public epitopes
Source: PLoS Pathog. 2025 Oct 21;21(10):e1013086. doi: 10.1371/journal.ppat.1013086 (PMC12551957; doi:10.1371/journal.ppat.1013086)

**S2 Fig.** Most HPV16 binding antibodies neutralize

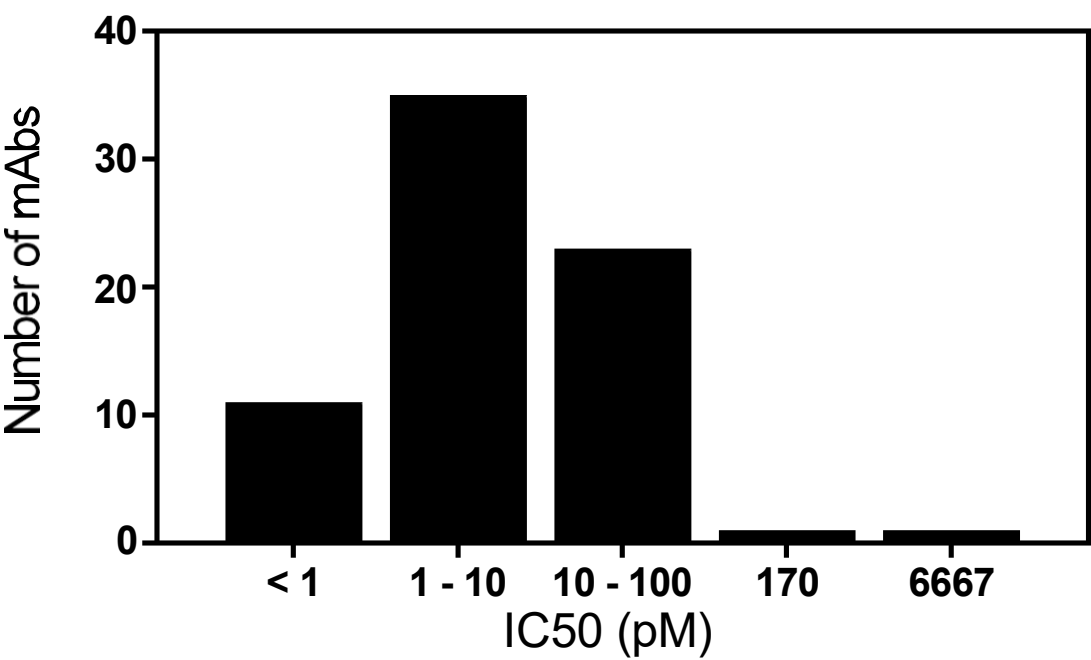

Supplement: S2 Fig — A histogram showing the distribution of HPV16 IC50 neutralization values for the 68 hmAbs used in this study. (PDF) [file ppat.1013086.s006.pdf]

**S3 Fig.** HPV16 L1 loops required for neutralization for each of four subjects.

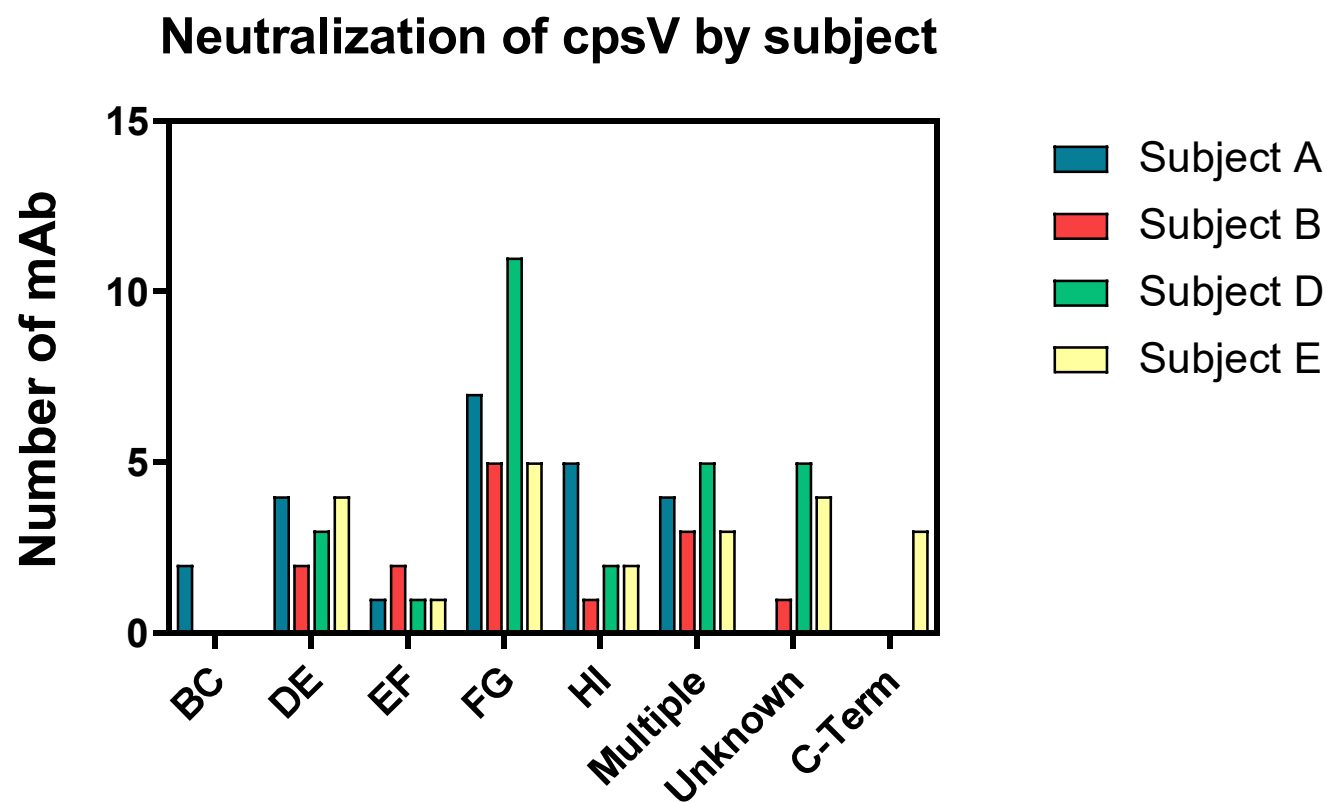

Supplement: S3 Fig — The number of mAb that required a particular loop (indicated on the horizontal axis), multiple loops or the c-terminus in either the primary or secondary tests is shown for each of four subjects (different colored bars). (PDF) [file ppat.1013086.s007.pdf]

S4 Fig. Neutralization profile of mAb that use IGKV1-39.

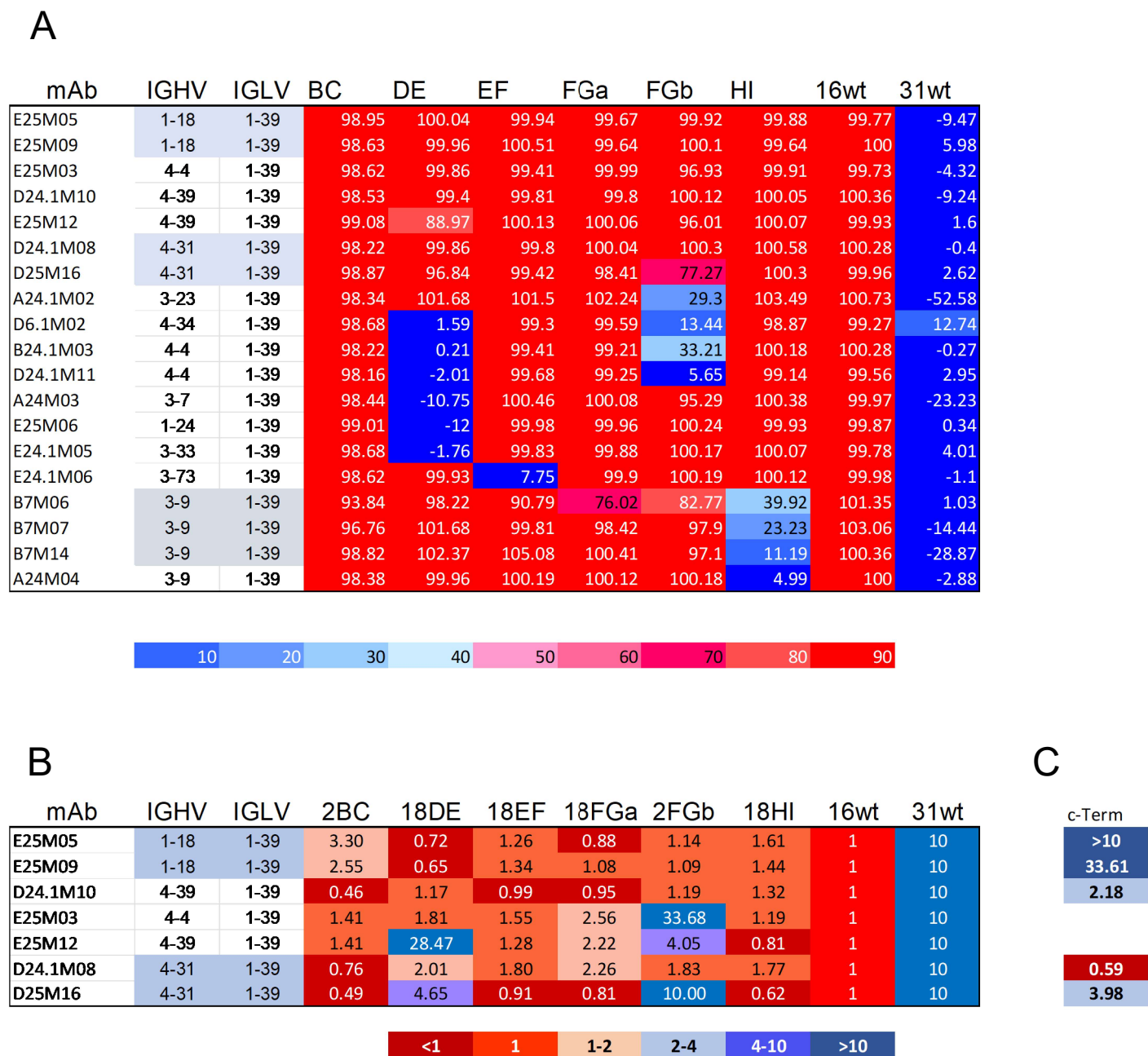

Supplement: S4 Fig — List of the percent neutralization for each mAb (left column) versus each cpsV (indicated on top) in the first screen (A) and the IGHVs for all mAb that used IGKV1–39. B) Results in fold difference in titer compared with psV16 for mAbs vs each cpsV (top). The fold difference titer vs cpsV with C-terminal amino acid substitutions is on the right, tested separately. A value of 10 was assigned when a mAb failed to neutralize at least 50%. These are the same data as in Fig 1 and S2 Table. (PDF) [file ppat.1013086.s008.pdf]

S5 Fig. Importance of CDRL2 for B25M02 neutralization

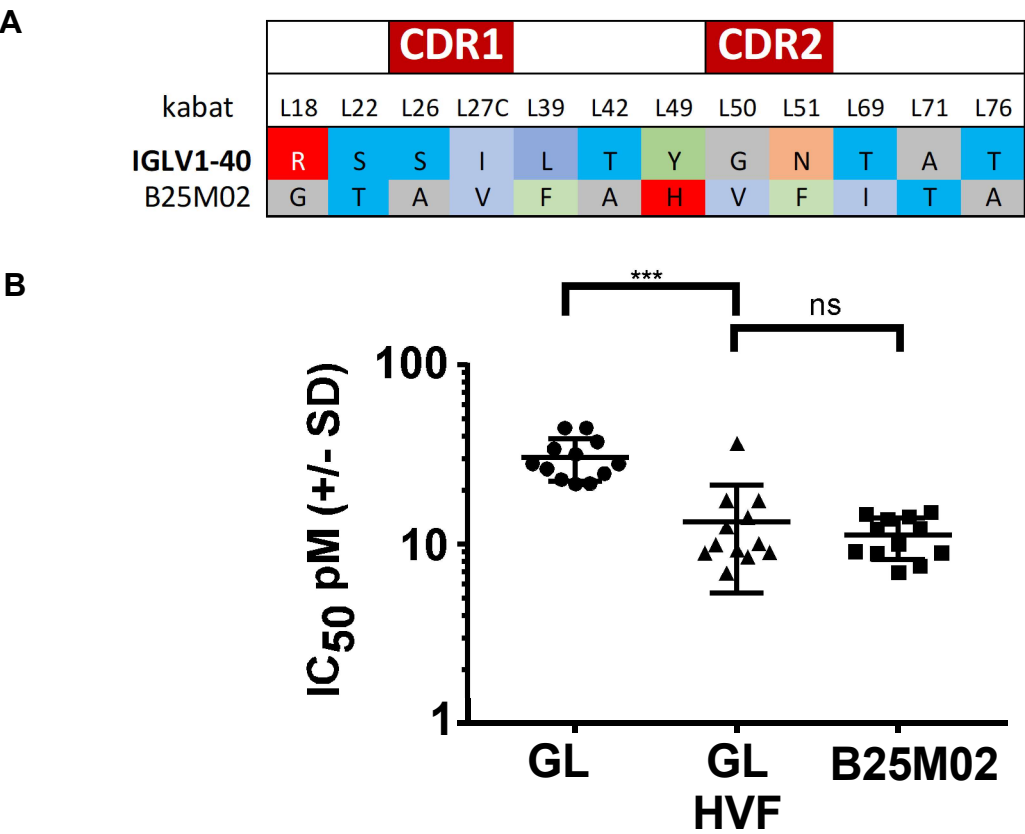

Supplement: S5 Fig — B25M02 light chain with just the CDRL2 mutated – see sequences – panel A. B25M02 heavy chain was coexpressed with either the fully mature B25M02 light chain, fully germline IGLVl1-40 light chain or the germline light chain with the mature (HVF) CDRL2. Each data point (B) is an IC50 vs psV16, determined from a titration curve. *** = p < 0.001. ns = not significant. GL = germline seq. (PDF) [file ppat.1013086.s009.pdf]

**S6 Fig.** Data processing of cryo-EM data

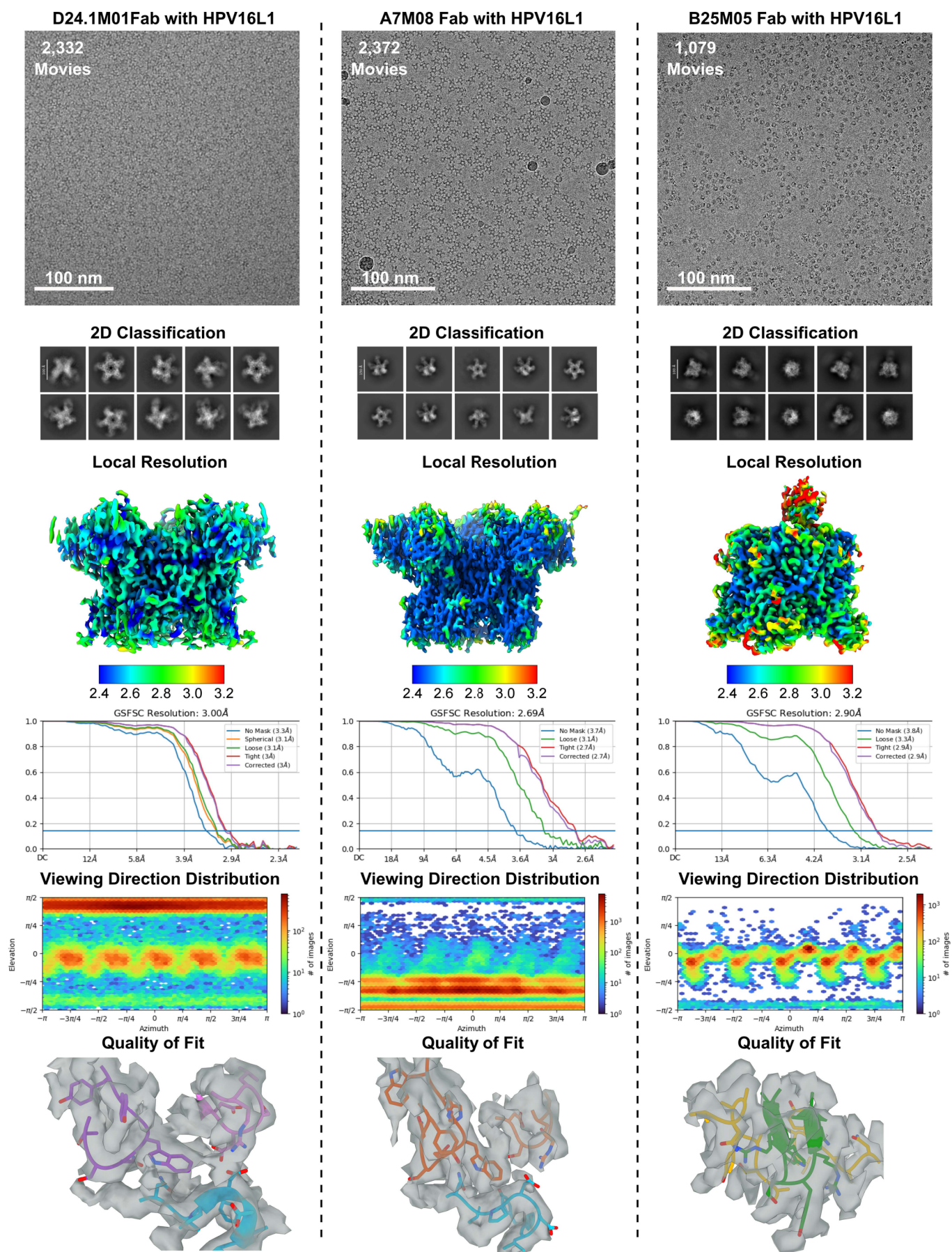

Supplement: S6 Fig — Cryo-EM reconstructions of D24.1M01 Fab bound to HPV16L1 (left), A7M08 Fab bound to HPV16L1 (middle), and B25M05 Fab bound to HPV16L1 (right). Representative micrographs, representative 2D class averages, final resolved map colored based on local resolution estimation, and FSC plot with resolution estimation based on GSFSC0.143, and view direction distribution are shown. A quality of fit image for each Fab at the binding interface with HPV16 L1 is shown. Only the cryo-EM reconstruction within 3 Å of the protein is shown for clarity. (PDF) [file ppat.1013086.s010.pdf]

S7 Fig. BSA Plots Antibodies

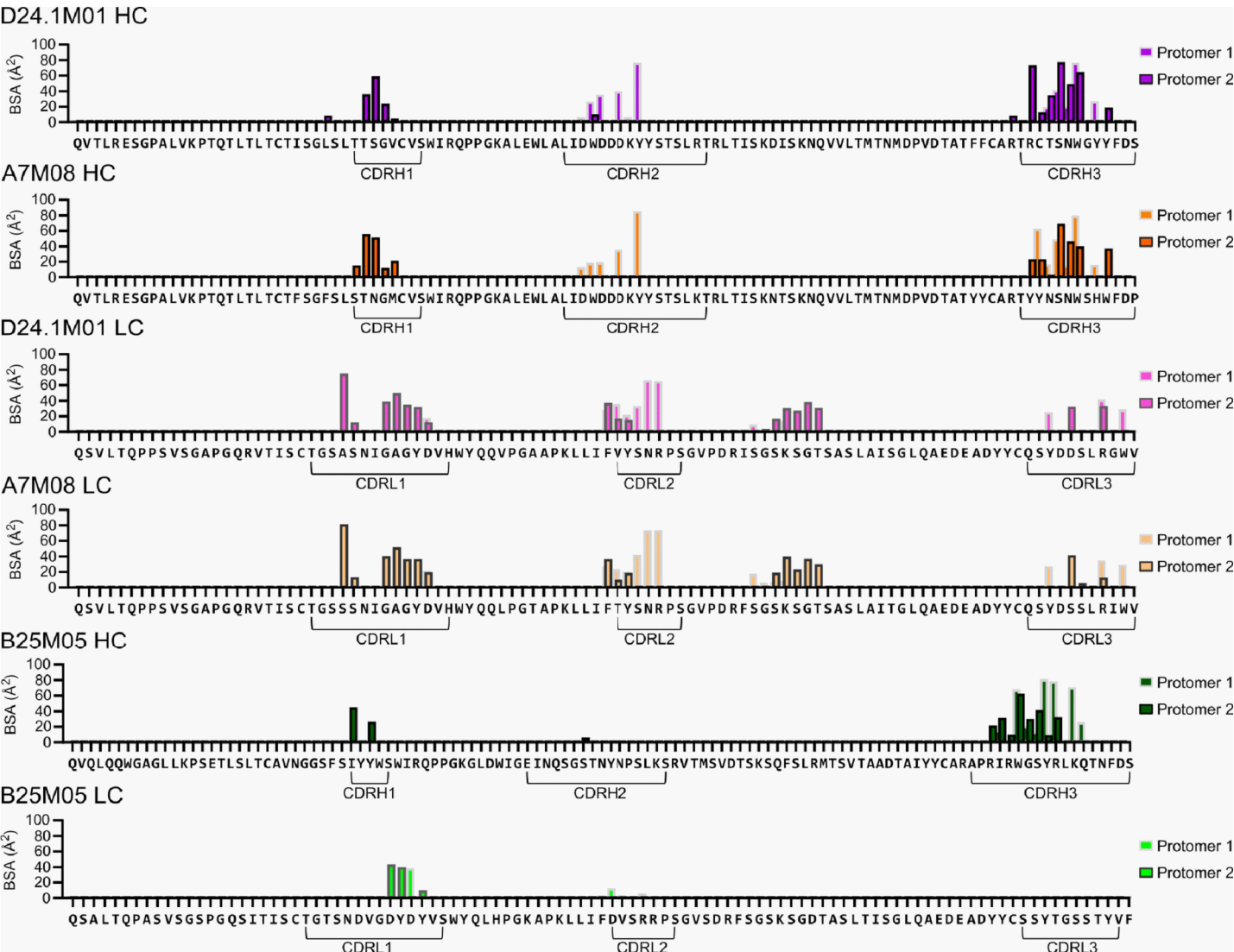

Supplement: S7 Fig — Buried surface area (BSA) of each mAb amino acid upon binding with HPV16 L1. Values were determined using the PDBePISA server [73]. Interactions with protomer 1 are outlined in gray and interactions with protomer 2 are outlined in black. (PDF) [file ppat.1013086.s011.pdf]

S8 Fig. BSA Plots Antibodies

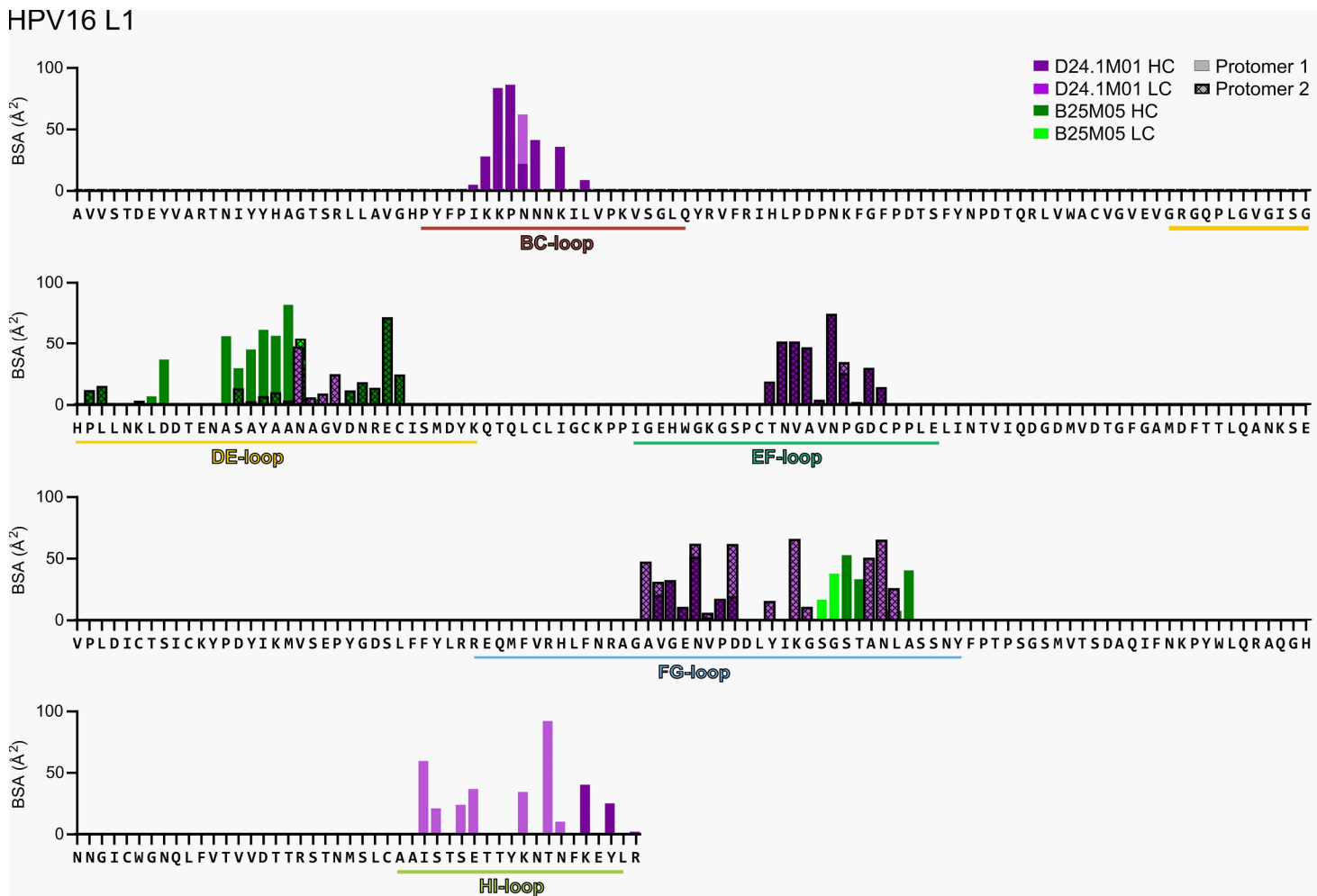

Supplement: S8 Fig — Buried surface area (BSA) of the amino acids of HPV L1 upon binding by D24.1M01 or B25M05. Values were determined using the PDBePISA server [73]. Heavy and light chain interactions are differentiated in dark and light colors. Amino acids from protomer 1 are solid colors and amino acids from protomer 2 have a checkered pattern. (PDF) [file ppat.1013086.s012.pdf]

**S9 Fig.** Importance of disulfide bond linking D24.1M01 CDRH1 and CDRH3 and movement of DE-loop in B25M05 binding

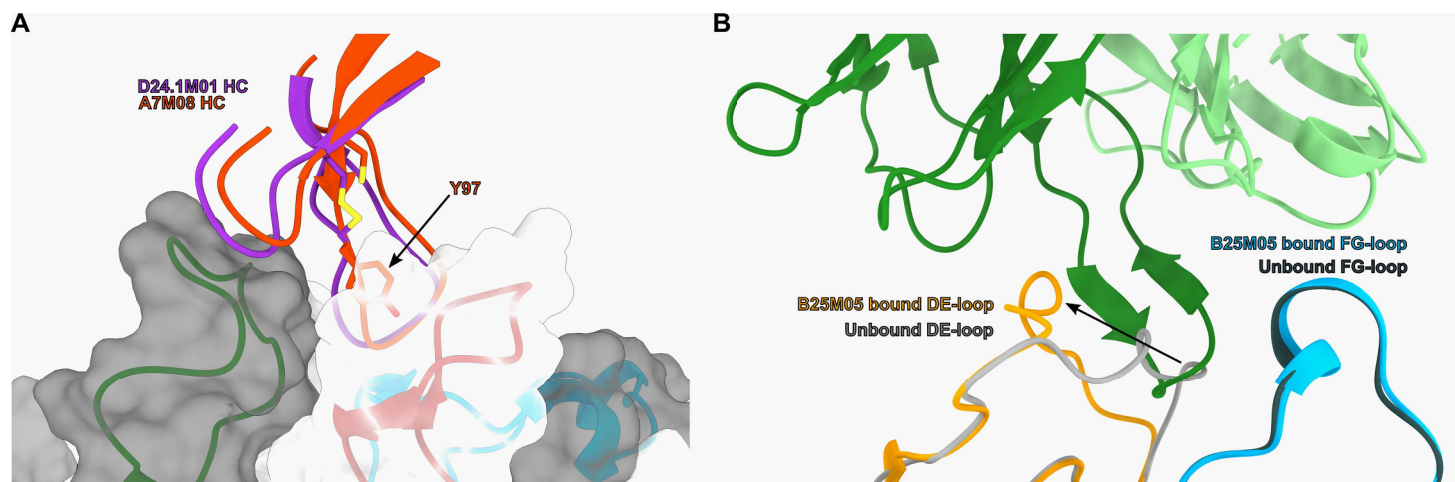

Supplement: S9 Fig — A) Cartoon diagram of the overlayed D24.1M01 and A7M08 structures. D24.1M01 has a disulfide bond linking the CDRH1 and CDRH3 but in A7M08 the CDRH3 contains a tyrosine. B) Cartoon diagram showing the movement of the DE-loop between unbound and B25M05 bound L1 protomers. Only the DE-loop undergoes rearrangement the FG-loop is unchanged upon binding. (PDF) [file ppat.1013086.s013.pdf]
